# Deep‐Learning‐Assisted Design of Dynamically Tunable Near‐Infrared Dual‐Band Lithium Niobate Metamaterial Absorber for Optical Communication

**DOI:** 10.1002/nap2.70262

**Published:** 2026-08-23

**Authors:** Xiajun Liu, Zexuan Zhang, Chenxi Su, Mei Wang, Feng Xia, Peng Sun, Li Dong, Maojin Yun

**Affiliations:** ^1^ College of Physics Qingdao University Qingdao China; ^2^ State Key Laboratory of Physical Oceanography Institute of Oceanographic Instrumentation, Qilu University of Technology (Shandong Academy of Sciences) Qingdao China

## Abstract

To overcome the fixed operating wavelengths and limited tunability of conventional metamaterial absorbers, this study proposes a dynamically tunable near‐infrared dual‐band lithium niobate (LN) metamaterial absorber platform at optical communication wavelengths. Using an “end‐to‐end + gradient optimization” deep learning inverse‐design framework, we efficiently and accurately derive the structural parameters matching target absorption spectra. The designed LN metamaterial absorber (LN‐MMA) exhibits dual‐band near‐perfect absorption peaks at 1310 and 1550 nm, achieving peak efficiencies of 99.9% and 99.5%, respectively. Owing to the strong electro‐optic effect of LN, the resonance wavelengths can be dynamically tuned via an external electric field. Furthermore, by integrating the LN‐MMA with coherent technology, a dynamically tunable coherent perfect absorber (LN‐CPA) is realized, exhibiting absorption peaks at 1550 and 1704 nm with efficiencies of 98.5% and 98.1%. In this configuration, the absorption response can be actively tuned by the phase difference between two incident coherent beams, enabling all‐optical modulation. These results demonstrate a compact absorber with both electrical and optical tunability, showing strong potential for inter‐satellite laser communication, optoelectronic signal conversion, optical detection, and all‐optical signal processing.

## Graphical abstract

An end‐to‐end deep‐learning framework combining inverse prediction and gradient refinement enables rapid design of dynamically tunable lithium‐niobate metamaterial absorbers for optical communication. Near‐perfect dual‐band absorption is achieved at 1310 and 1550 nm with electro‐optic wavelength tuning. Coherent illumination further produces near‐perfect absorption at 1550 and 1704 nm, enabling phase‐controlled all‐optical modulation in a compact platform.

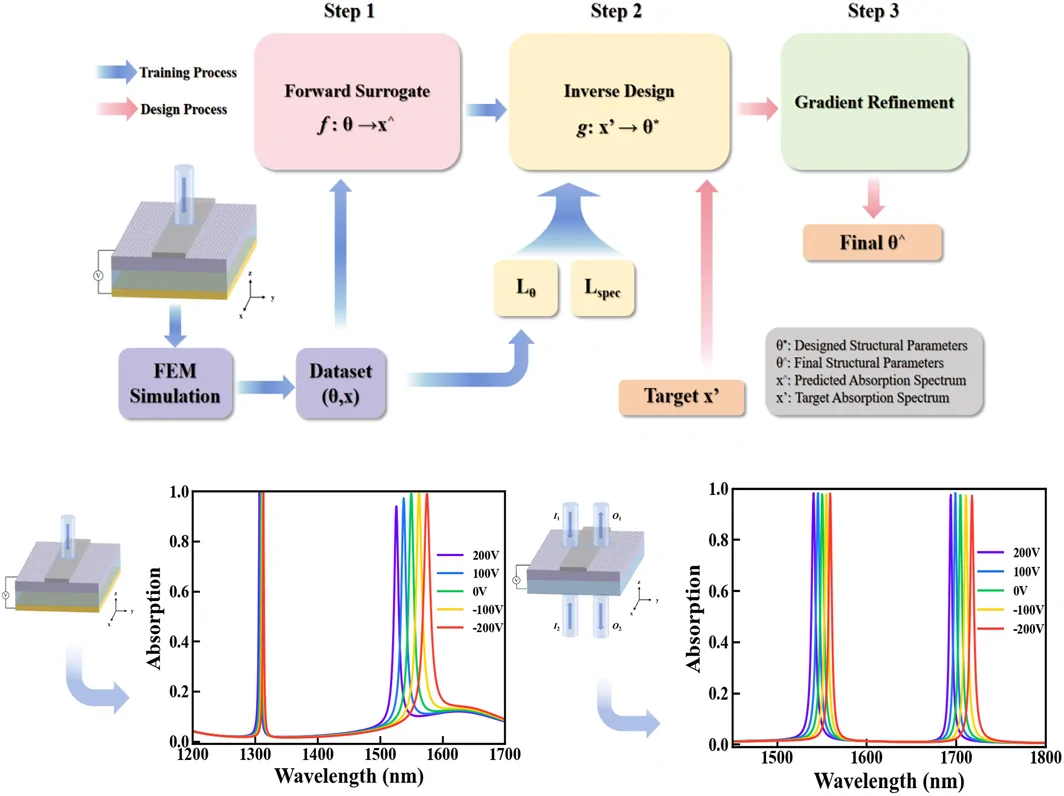

## 1 Introduction

Inter‐satellite laser communication offers high transmission rates, confidentiality, compact size, and low power consumption, making it ideal for long‐distance, high‐speed information transmission [1, 2]. However, the signal can be disrupted by the stray light, so efficiently absorbing stray light in the optical path is crucial [3]. Metamaterials, artificial electromagnetic media with exotic responses such as negative refractive index and perfect absorption [4, 5], offer a promising solution. Since the first demonstration of a metamaterial absorber (MMA) by Landy et al. in 2008, MMAs have been extensively developed across various bands [6, 7, 8, 9, 10, 11, 12, 13]. Nevertheless, most reported MMAs operate at fixed wavelengths, which severely limits their applicability in dynamically reconfigurable photonic systems.

To achieve tunability, functional materials such as graphene, liquid crystals, and vanadium dioxide have been explored in MMAs [14, 15, 16, 17], whereas recent all‐silicon terahertz metasurfaces have demonstrated an alternative approach based on mechanical rotation, enabling tunable focusing and polarization control through Moiré and spin‐decoupled phase engineering [18, 19]. Lithium niobate (LN), a widely used electro‐optic (EO) material in integrated optics [20, 21, 22, 23], is an attractive alternative due to its strong Pockels effect. Beyond EO tuning in single‐port configurations, the coherent perfect absorber (CPA) proposed by Chong et al. offers an all‐optical mechanism for dynamically controlling absorption [24]. By exploiting the coherent interference of two counter‐propagating beams in a well‐designed structure, CPA allows absorption to be tuned from nearly 0% to 100% by adjusting their relative phase [25, 26]. CPA has been widely explored for optical detection and signal processing applications [27, 28].

Despite these advances, realizing high‐performance multiband LN‐based MMAs or CPAs remains challenging, primarily due to the large, strongly coupled design space of their structural parameters. Conventional trial‐and‐error parameter sweeping methods are time‐consuming and computationally expensive, making them unsuitable for systematic multiband design. This highlights the need for rapid and accurate inverse design methods. In recent years, deep learning techniques, including multilayer perceptrons (MLP), convolutional neural networks (CNN), and Transformers, have shown great potential in the inverse design of metasurfaces [29, 30, 31]. However, building accurate mappings between spectral responses and metasurface parameters, particularly for narrowband absorption peaks, remains challenging. High‐performance models such as residual MLP (ResMLP) and Kolmogorov–Arnold networks (KAN) [32, 33, 34], together with tailored network frameworks, are crucial for further enhancing design accuracy and capturing subtle spectral features.

In this work, we propose a dynamically tunable near‐infrared LN‐MMA and establish an “end‐to‐end + gradient optimization” deep learning inverse‐design framework to retrieve its structural parameters. The absorber achieves dual‐band near‐perfect absorption at 1310 and 1550 nm, with peak efficiencies of 99.9% and 99.5%, respectively, and its resonance wavelengths can be dynamically tuned via the EO effect of LN. Furthermore, by integrating the LN‐MMA with coherent absorption technology, we design a dynamically tunable coherent perfect absorber (LN‐CPA), whose structural parameters are also obtained via the proposed deep learning framework. The LN‐CPA exhibits near‐perfect absorption at 1550 and 1704 nm with efficiencies of 98.5% and 98.1%, and offers an additional all‐optical tuning mode by adjusting the phase difference between two incident coherent beams. By combining deep learning‐based inverse design with functional EO materials, this work demonstrates a compact absorber with dual electrical and optical tunability, showing broad application prospects in inter‐satellite laser communication, optoelectronic signal conversion, optical detection, and all‐optical signal processing.

## 2 MMA Inverse Design Using Deep Learning

### 2.1 Structure and Simulation Methods

The unit cell of the proposed metamaterial absorber (Figure 1) consists of five layers, listed from top to bottom: a grating layer (refractive index *n*_1_ = 1.5, thickness *h*_0_ = 200 nm, width *w*), monolayer graphene, LN (thickness *h*_1_), a transparent dielectric layer (refractive index *n*_2_ = 1.45, thickness *h*_2_), and Au substrate (thickness *h*_3_). This planar multilayer structure could be fabricated through sequential Au and dielectric deposition, thin‐film LN transfer or bonding, graphene transfer, and top‐grating patterning using established micro‐ and nanofabrication techniques [35, 36, 37]. The period is *P*_*x*_ = *P*_*y*_ = 1000 nm. Here, *h*_1_, *h*_2,_ and *w* are used as tunable parameters for inverse design. Additionally, *h*_0_ is fixed to simplify the grating geometry, whereas *h*_3_ is set sufficiently large to suppress transmission. Thus, the absorption behavior is mainly determined by *h*_1_, *h*_2,_ and *w*, which are used for dataset construction in Section 2.2. All results are obtained from finite element method (FEM) simulations, with periodic boundary conditions along the *x*‐ and *y*‐axes and a perfectly matched layer along *z*‐axis. Graphene is modeled as a conductive surface with an optical conductance *G*_0_ = *e* ^2^/(4*ℏ*) ≈ 6.08 × 10^−5^ Ω^−1^, corresponding to 2.3% absorption for a free‐standing film [38]. The inclusion of graphene allows the metasurface to satisfy the waveguide matching conditions, thereby facilitating strong light–matter interaction and significantly enhancing absorption. When a voltage bias is applied, the refractive index of LN can be described as [39]

(1) $$n_{x}=n_{y}=n_{o}-\frac{n_{o}^{3}r_{13}E_{z}}{2},$$

(2) $$n_{z}=n_{e}-\frac{n_{e}^{3}r_{33}E_{z}}{2},$$

where *r*_13_ = 8.5 pm/V and *r*_33_ = 28.5 pm/V are the EO coefficients of LN and *E*_*z*_ is the electric field intensity along the *z*‐direction [40]. The ordinary and extraordinary absolute refractive indices *n*_*o*_ and *n*_*e*_ are given by [41]

(3) $$n_{o}^{2}=4.9048-\frac{0.11768}{0.04750-\lambda^{2}}-0.027169\lambda^{2},$$

(4) $$n_{e}^{2}=4.5820-\frac{0.099169}{0.044432-\lambda^{2}}-0.021950\lambda^{2},$$

where *λ* (μm) is the wavelength of the incident light. According to Equations ((1), (2), (3), (4)), the refractive index of LN varies with the applied voltage, achieving a dynamically tunable MMA structure.

**FIGURE 1:**
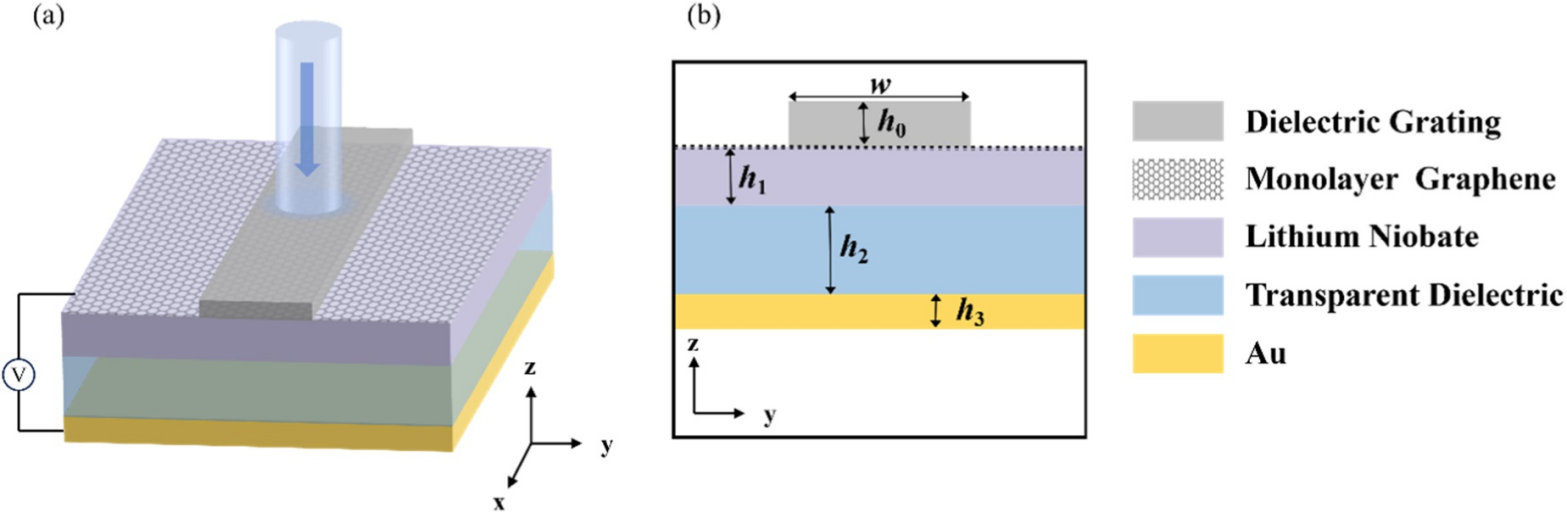
(a) Schematic of the proposed LN metamaterial absorber. (b) Cross‐section view of the proposed LN metamaterial absorber.

### 2.2 Deep‐Learning‐Assisted Design of the MMA

To enable an efficient inverse design of the metamaterial absorber, we first constructed a dataset using three structural parameters, *h*_1_, *h*_2,_ and *w*, sampled by Latin hypercube within *h*_1_ ∈ [100, 200] nm, *h*_2_ ∈ [1100, 1200] nm, and *w* ∈ [500, 600] nm. For each sample, the corresponding absorption spectrum was obtained via finite‐element‐method (FEM), yielding 5000 data samples. The spectra were sampled over the wavelength range from 1200 to 1700 nm with a step size of 2 nm. The dataset was randomly split into training, validation, and test sets in an 8:1:1 ratio (4000/500/500). Before training, both the input spectra and the output structural parameters were normalized to improve numerical stability and facilitate model convergence. All experiments were conducted on a Windows 10 system (build 10.0.22621) using Python 3.9.7, equipped with an Intel Core i9‐12900H CPU and 32 GB RAM, and all timing results reported in this work were obtained under CPU‐only execution. The deep‐learning design process follows an “end‐to‐end + gradient optimization” framework (Figure 2) with three steps:

**FIGURE 2:**
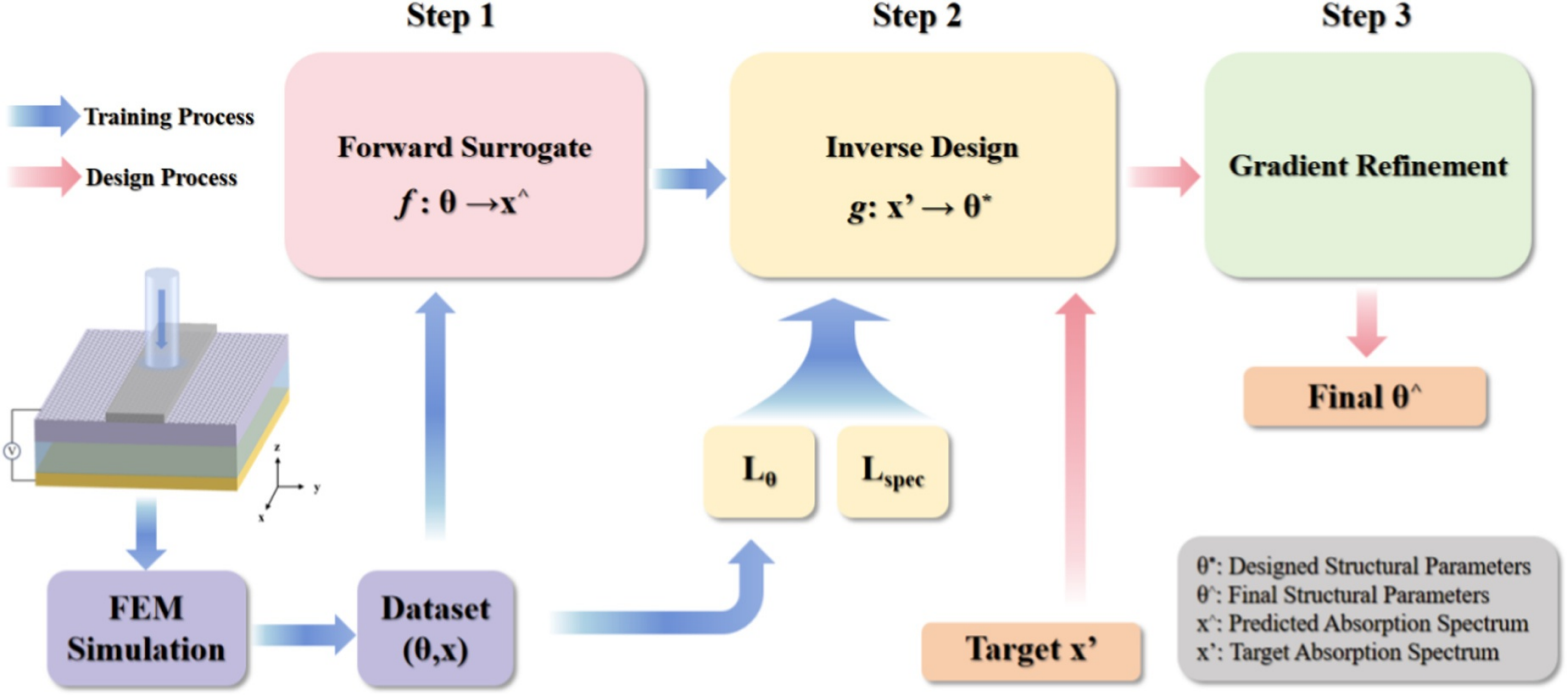
Deep learning‐based design framework.

Step 1: Forward Surrogate. A fast surrogate model *f* is built to map structural parameters *θ* to the predicted spectrum *x^*, serving as the “teacher” for inverse design. The forward model adopts a ResMLP architecture with six residual blocks (Figure 3). The multilayer residual structure enhances feature extraction and alleviates vanishing gradients. The model is trained using mean squared error (MSE):

(5) $$\text{MSE}=\frac{\sum {\left(x-x^{\wedge }\right)}^{2}}{N},$$

where *x* denotes the truth spectrum, and *x^* is the predicted spectrum obtained by the forward surrogate model, and *N* is the sample size.

**FIGURE 3:**
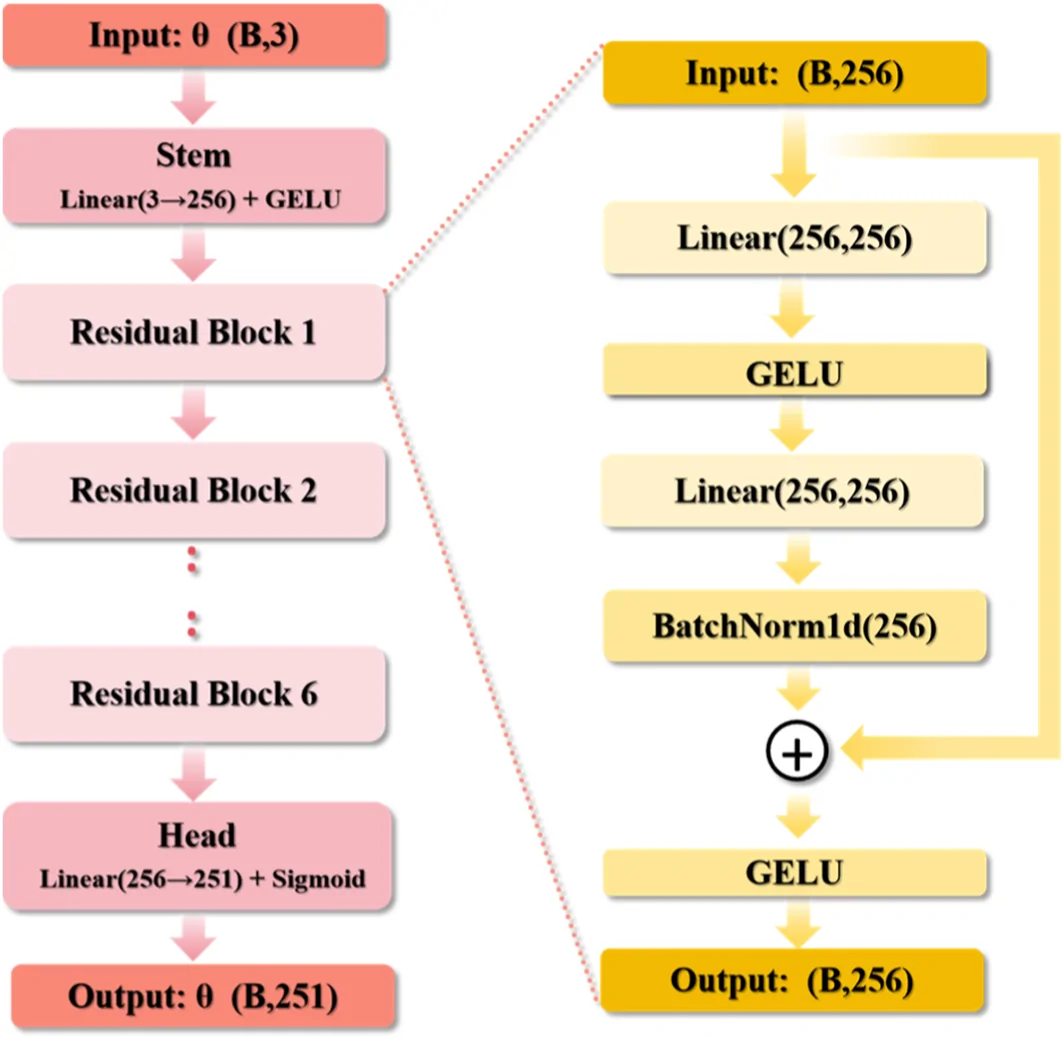
Architecture of the forward surrogate model based on ResMLP.

Step 2: Inverse Design. An inverse network *g* is built to map a target spectrum $x^{\prime }$ to the structural parameters $\theta^{*}$. The network was implemented as a fully connected KAN. To impose physical consistency during training, *g* was cascaded with the pretrained and frozen forward surrogate *f*, yielding a differentiable closed loop, $x^{\prime }$ → $\theta^{*}$ → *x* ^*^*^ = *f* ($\theta^{*}$). The inverse model was optimized using a composite loss *L*_inv_ = *L*_*θ*_ + *L*_spec_, where *L*_*θ*_ penalizes deviations from the labeled structural parameters and *L*_spec_ minimizes the mismatch between the reconstructed and target spectra. This design allows the inverse model to generate stable and physically meaningful initial solutions for subsequent gradient‐based refinement.

Step 3: Gradient Refinement. After training, the target spectrum $x^{\prime }$ was constructed according to the desired absorption response. The target spectrum is constructed as a superposition of multiple Gaussian peaks. When the target spectrum includes two absorption peaks, it is defined as

(6) $$x^{\prime }\left(\lambda \right)=\max\left(G_{1}\left(\lambda ;\mu_{1},\sigma_{1}\right),G_{2}\left(\lambda ;\mu_{2},\sigma_{2}\right),\text{baseline}\right),$$

where *G*_1_ and *G*_2_ represent the two Gaussian peaks, respectively, and the baseline value is typically set to a small constant, such as 0.05. The target spectrum was then fed into the trained inverse network to obtain an initial structural design, $\theta^{*}$ = *g* ($x^{\prime }$). Starting from $\theta^{*}$, gradient descent was performed with the pretrained and frozen forward surrogate *f* to further optimize the structural parameters by minimizing the spectral mismatch. Through iterative optimization, the final optimal parameters θ^ are obtained. Steps 2 and 3 are illustrated in Figure 4. The training settings for the three stages are summarized in Table 1.

**FIGURE 4:**
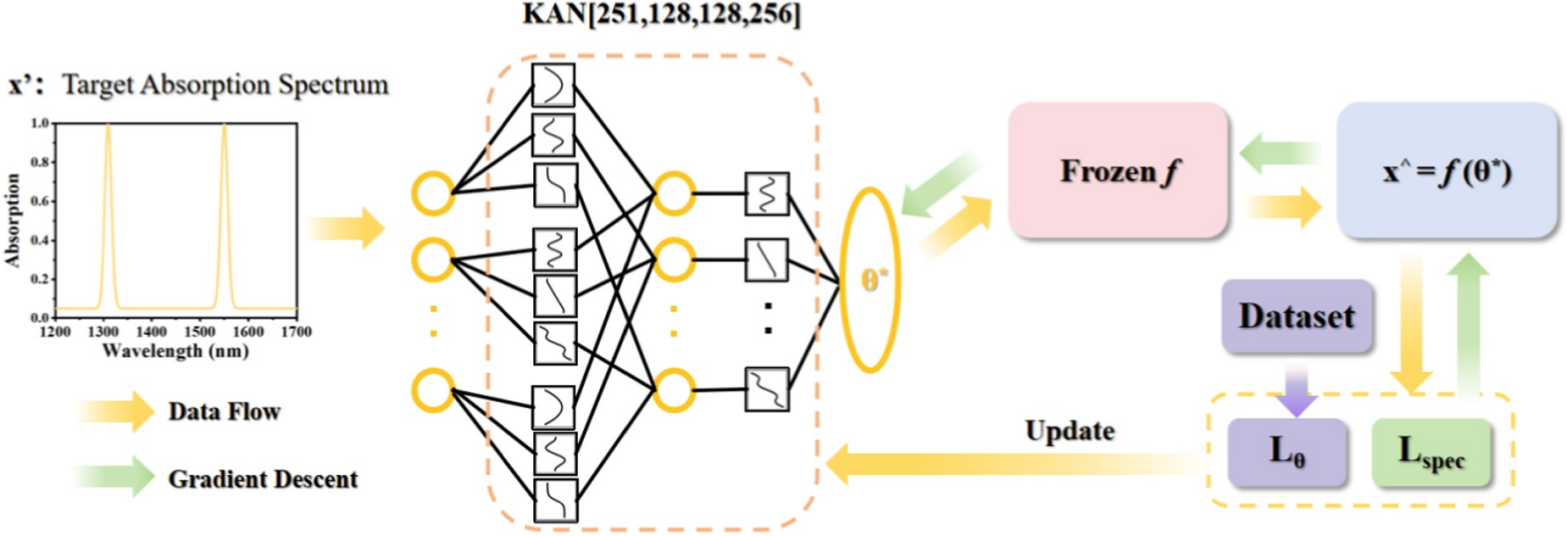
Inverse design and gradient refinement process.

**TABLE 1:** Training settings of the proposed deep‐learning framework.

Setting	Forward surrogate	Inverse model	Gradient refinement
Optimizer	AdamW	AdamW	Adam
Initial learning rate	1 × 10^−3^	1 × 10^−3^	1 × 10^−2^
Weight decay	1 × 10^−6^	1 × 10^−6^	—
Batch size	32	32	—
Learning‐rate scheduler	Cosine annealing	Cosine annealing	—

## 3 Results and Discussion

### 3.1 Design Analysis

The training performance of the forward surrogate model is summarized in Figure 5. After 120 epochs, the training loss (2.88 × 10^−4^) and validation loss (2.93 × 10^−4^) are nearly identical, indicating consistent performance on the sampled data distribution without observable overfitting. As shown in Figure 5b,c, our ResMLP‐based surrogate achieves significantly higher spectral reconstruction accuracy than conventional MLP and CNN models, especially in reconstructing peak regions. Compared with traditional FEM and finite‐difference time‐domain (FDTD), the proposed model avoids repeatedly solving Maxwell's equations and directly predicts metasurface absorption from trained data.

**FIGURE 5:**
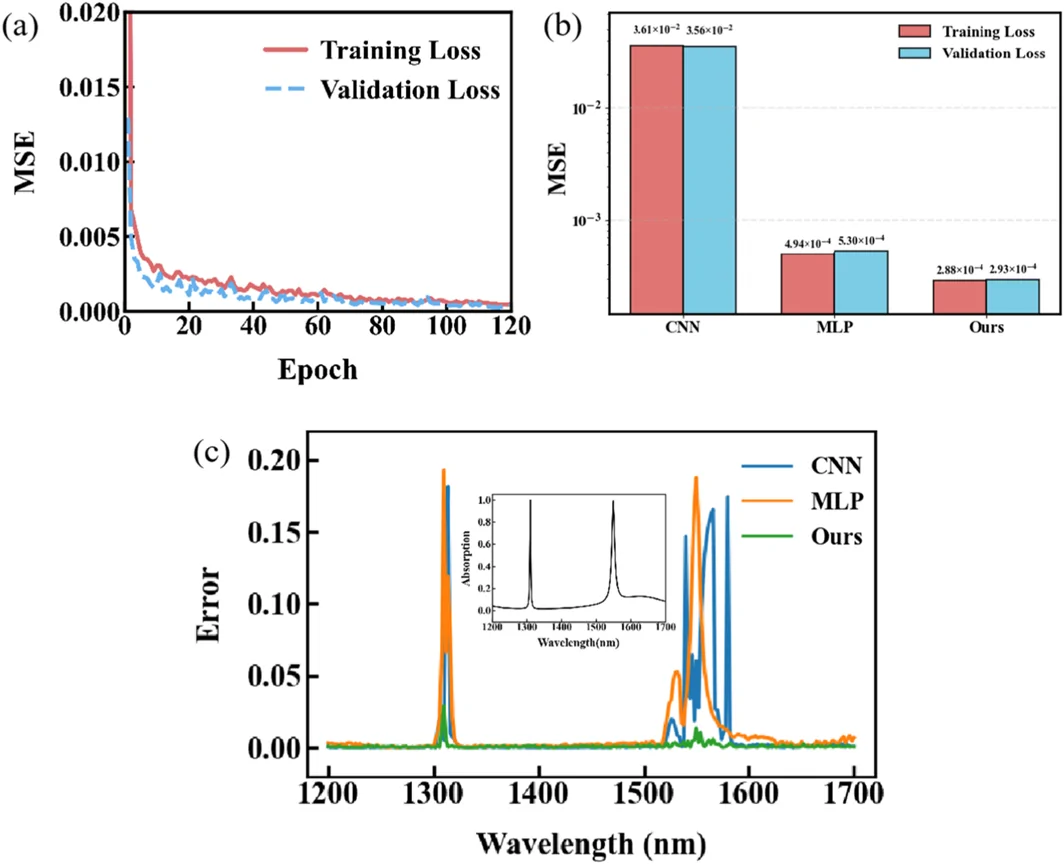
Training performance of the forward surrogate model. (a) The training and validation loss curves during the training process. (b) Loss values for different algorithms, including CNN, MLP, and our proposed method. (c) Prediction errors for different methods across the wavelength range. The inset shows the actual absorption spectrum.

Next, we evaluated the inverse design and gradient optimization stages for a dual‐band absorber targeting perfect absorption at 1310 and 1550 nm, where the target spectrum is constructed according to Equation (6). As shown in Figure 6a, the total loss rapidly decreases and converges to a stable value, with the final training and validation losses of 1.61 × 10^−2^ and 1.62 × 10^−2^, remaining close. This behavior indicates stable optimization and consistent performance on the training and validation datasets. To further quantitatively assess the contribution of different model components, an ablation study is conducted by comparing three configurations: an MLP‐based inverse model without gradient refinement (MLP‐NR), a KAN‐based model without gradient refinement (KAN‐NR), and our full KAN‐based model with gradient refinement (Ours). The structural parameters for each design are listed in Table 2. The absorption spectra in Figure 6b show that our approach achieves more accurate dual‐peak reconstruction and improved peak‐position control compared with the baseline models. These results clearly demonstrate both the superior capability of KAN in modeling strongly nonlinear spectrum–structure relationships and the critical role of gradient‐based refinement in correcting residual spectral mismatches, particularly for narrowband resonances. Overall, the proposed framework enables accurate target‐spectrum design within the sampled parameter domain. Its reliable application to a wider design space can be further improved by expanding the FEM dataset and retraining the model.

**FIGURE 6:**
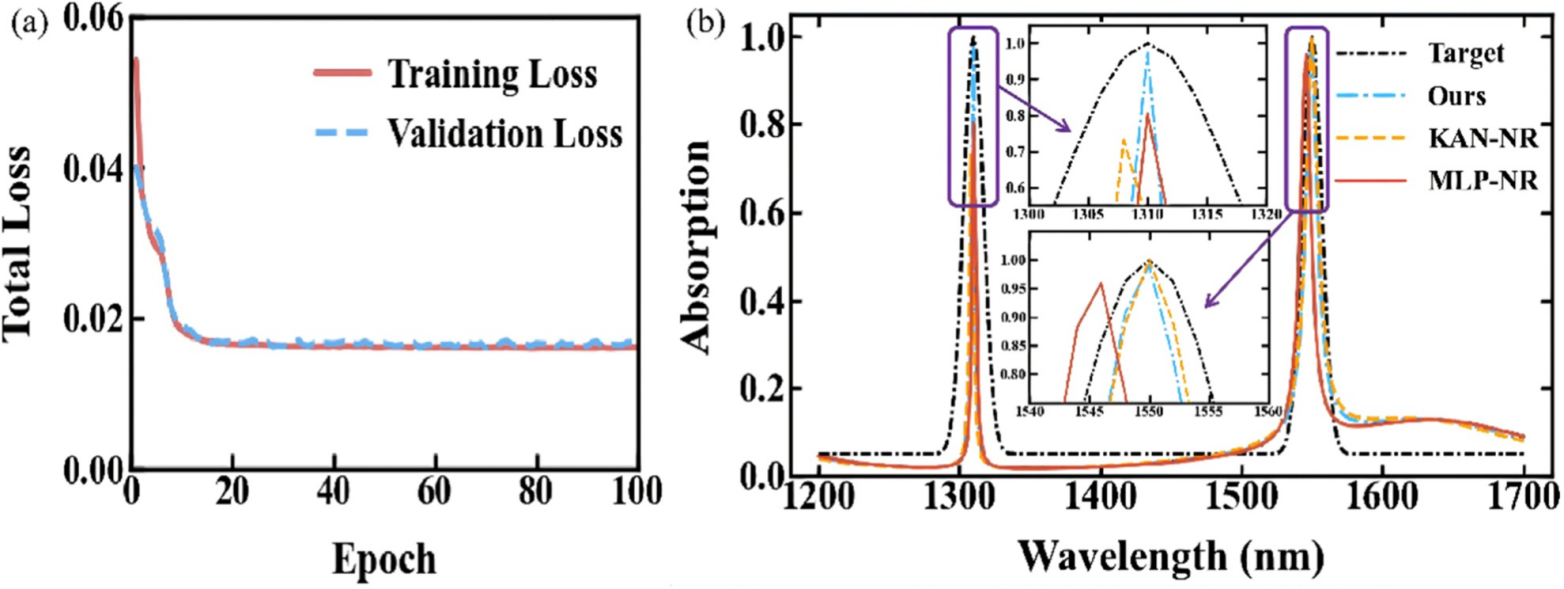
(a) Total loss of the inverse design model. (b) Ablation experiment: Absorption spectra corresponding to different MMA design schemes.

**TABLE 2:** Results of different MMA design schemes.

Inverse design solution	*h*_1_ (nm)	*h*_2_ (nm)	*w* (nm)
MLP‐NR	137.41	1123.05	557.01
KAN‐NR	140.37	1109.43	570.12
Ours	140.01	1115.05	570.02

### 3.2 MMA Analysis

The absorption performance of the MMA is analyzed based on the design described above. As shown by the green absorption curve in Figure 7a, under *x*‐polarized incidence at a bias voltage of 0 V, the absorber exhibits two near‐perfect absorption peaks at *p*_1_ = 1310 nm and *p*_2_ = 1550 nm, with efficiencies of 99.9% and 99.5%, respectively. These two resonances correspond to the target wavelengths used in the inverse‐design process, confirming the accuracy of the proposed framework. The absorption properties can be dynamically tuned by an applied voltage. When a forward voltage is applied, the absorption peaks exhibit a blue shift. Conversely, the absorption peaks exhibit a red shift under a reverse voltage. As shown in Figure 7b, linear fitting yields voltage modulation rates of −0.015 nm/V and −0.123 nm/V for the first and second peaks, respectively. This can be explained using the following equation [42]:

(7) $$f=\frac{\varphi_{p}c}{4h_{1}\sqrt{\varepsilon_{r}-\sin^{2}\;\alpha }},$$

where *f*, *φ*_*p*_, and *c* are the resonance frequency, propagation phase, and speed of light in free space, respectively. In our simulation, the thickness of the LN layer *h*_1_ and incident angle *α* are fixed values, and *φ*_*p*_ is also considered to be a fixed value because the incident wave is a plane wave. According to Equations (1) and (2), when a forward voltage is applied, the effective refractive index of LN decreases, and the effective dielectric constant of LN *ε*_*r*_ decreases. As a result, Equation (7) shows a blue shift resulting from an increase in resonance frequency. Similarly, a reverse voltage has the opposite effect, leading to a red shift. Therefore, the designed LN metamaterial absorber has good voltage‐wavelength discrimination at 1310 and 1550 nm, demonstrating significant potential for applications in voltage detection, sensing, and photoelectric signal conversion. The ± 200 V range in Figure 7 is used to illustrate the electro‐optic tuning trend. A device‐level voltage of 200 V has been experimentally applied in a related vertically biased thin‐film lithium‐niobate multilayer device [43]. In the present structure, the graphene and Au layers could serve as the top and bottom electrodes, respectively. Since electro‐optic modulation is governed by the electric‐field strength rather than the applied voltage itself, the practical operating voltage depends on the electrode spacing, the local electric‐field distribution, and the breakdown limits of the multilayer stack. Lower‐voltage operation may be achieved by optimizing the electrode geometry, reducing the electrode spacing, enhancing the overlap between the electric field and the optical mode, and increasing the electro‐optic sensitivity of the resonance [44].

**FIGURE 7:**
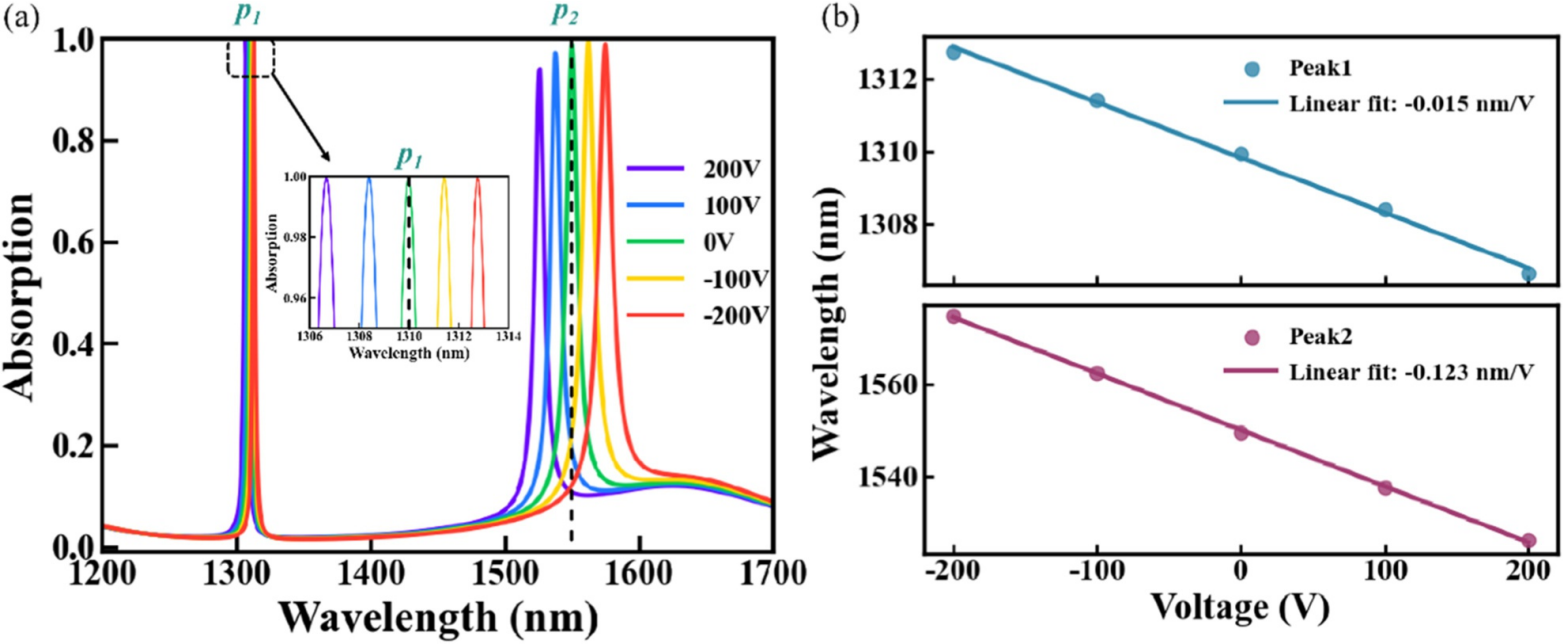
(a) Absorption spectra of the LN metamaterial absorber with different applied voltages. (b) Absorption peaks of the LN metamaterial absorber versus voltage with corresponding linear fits.

To investigate the physical origin of the two near‐perfect absorption peaks at *p*_1_ = 1310 nm and *p*_2_ = 1550 nm, we analyze their electric field distributions, as shown in Figure 8. The electric field of the *p*_1_ peak is mainly concentrated in the transparent dielectric layer, whereas the electric field of the *p*_2_ peak is mainly concentrated in the LN layer. To quantitatively evaluate the interaction between the resonant modes and the EO‐active LN layer, we calculated a 2D LN electric‐field overlap factor from the *y*‐*z* cross‐sectional field distributions, $\Gamma_{\mathrm{LN}}={\iint_{\mathrm{LN}}\left|E\left(y,z\right)\right|}^{2}dA/\iint_{\text{all}}{\left|E\left(y,z\right)\right|}^{2}dA$. The calculated $\Gamma_{\mathrm{LN}}$ values are 3.27% and 27.37% for the 1310 and 1550 nm resonances, respectively. This indicates that the stronger voltage tuning of the 1550 nm resonance mainly originates from its larger field overlap with the LN layer, whereas the weak tuning of the 1310‐nm resonance is caused by its finite evanescent‐field penetration into the LN layer. In both cases, the field profiles exhibit periodic distributions along the *y*‐direction, which is characteristic of quasi‐guided mode resonance [45, 46]. According to slab waveguide theory, transverse electric (TE) and transverse magnetic (TM) quasi‐guided modes should satisfy the following eigenvalue equations [47, 48]:

(8) $$\tan\left(\kappa d\right)=\frac{\kappa \left(\gamma_{c}+\gamma_{s}\right)}{\kappa^{2}-\gamma_{c}\gamma_{s}}\left(\text{TE}\;\text{mode}\right),$$

(9) $$\tan\left(\kappa d\right)=\frac{\varepsilon_{w}\kappa \left(\varepsilon_{s}\gamma_{c}+\varepsilon_{c}\gamma_{s}\right)}{\varepsilon_{c}\varepsilon_{s}\kappa^{2}-\varepsilon_{w}^{2}\gamma_{c}\gamma_{s}}\left(\text{TM}\;\text{mode}\right),$$

where *d* is the thickness of the waveguide layer, $\kappa =\sqrt{\varepsilon_{w}k_{0}^{2}-\beta^{2}}$, $\gamma_{c}=\sqrt{\beta^{2}-\varepsilon_{c}k_{0}^{2}}$, $\gamma_{s}=\sqrt{\beta^{2}-\varepsilon_{s}k_{0}^{2}}$, *k*_0_ is the wavenumber in free space, and *ɛ*_*w*_, *ɛ*_*c*_, and *ɛ*_*s*_ are the permittivities of the waveguide, the cladding, and the substrate layers, respectively. *β* is the propagation constant of the guided modes in the *x‐y* plane, $\beta =k_{x-y}+i\frac{2\pi }{P_{x}}x+j\frac{2\pi }{P_{y}}y$, where *k*_*x‐y*_ is the component of the incident wave vector in the *x‐y* plane, *i* and *j* are the integers labeling the orders of the diffracted waves in the *x*‐ and *y*‐axes, respectively. Considering normal incidence (*k*_*x‐y*_ = 0) and the first‐order grating vector, *β* can be simplified as *β* = 2π/*P* (*P* = *P*_*x*_ = *P*_*y*_ = 1000 nm). The refractive‐index distribution of the grating layer and the finite conductivity of graphene modify the effective modal index, field confinement, and radiation coupling are not included in this simplified expression for *β*, although both are considered in the FEM simulations. The waveguide layer corresponding to the absorption peak at *p*_1_ was a transparent dielectric layer. By bringing various parameters into Equations (8) and (9), the theoretical resonant wavelength of the TE quasi‐guided modes can be obtained as *λ*_1_ = 1319 nm. Similarly, the waveguide layer corresponding to the absorption peak at *p*_2_ is the LN layer, and the theoretical resonant wavelength of the TE quasi‐guided modes is obtained as *λ*_2_ = 1539 nm. This simplified treatment may account for the deviations of 9 nm (0.69%) and 11 nm (0.71%) from the simulated results. Despite these small deviations, the theoretical results, together with the electric‐field distributions in Figure 8, support the conclusion that the two absorption peaks at *p*_1_ and *p*_2_ originate from the TE quasi‐guided mode resonance supported by different waveguide layers.

**FIGURE 8:**
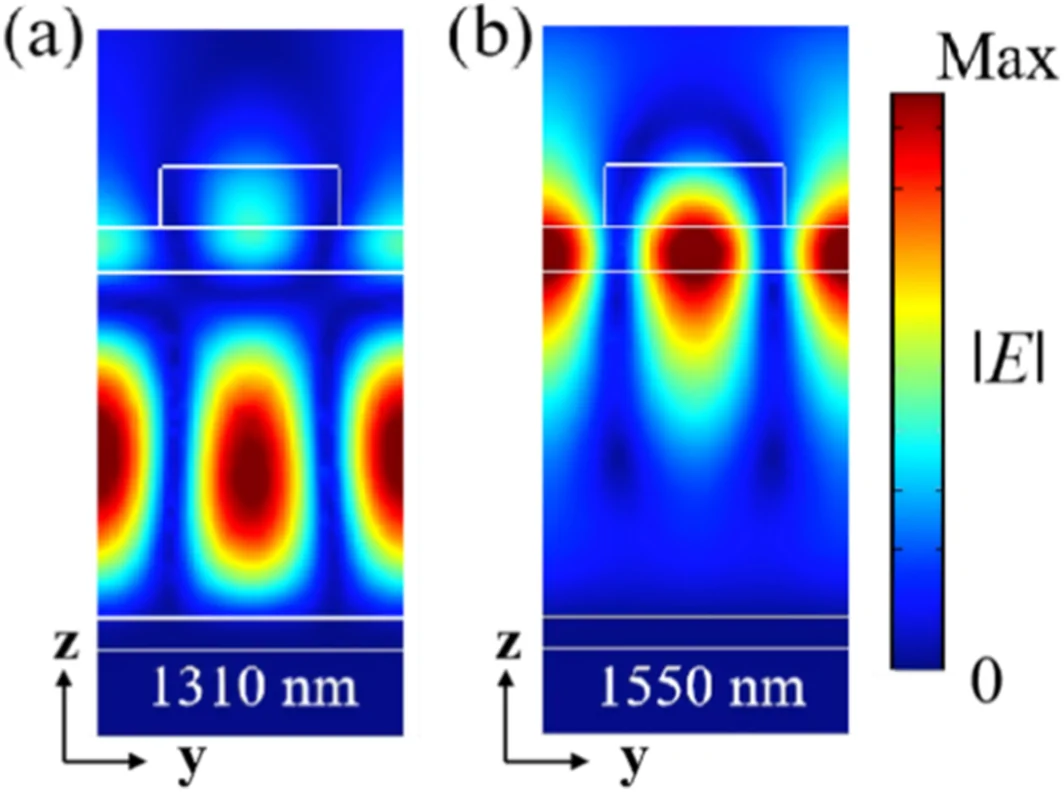
(a) Electric field distribution on the cross‐section view of the LN metamaterial absorber at 1310 nm. (b) Electric field distribution on the cross‐section view of the LN metamaterial absorber at 1550 nm.

Furthermore, the influence of the polarization angle on the absorption performance was investigated, as shown in Figure 9. The absorption performance remained at a high level when the polarization angle was increased from 0° to 60°, whereas the absorption peaks at 1310 and 1550 nm gradually weaken and eventually disappear as the polarization angle increased from 60° to 90°. Meanwhile, for polarization angles exceeding 15°, an additional absorption peak appears at 1374 nm, reaching a maximum absorptivity of 62% for *y*‐polarized incidence. Thus, the proposed LN‐MMA maintains strong absorption performance over a wide range of polarization angles, demonstrating a certain degree of polarization tolerance.

**FIGURE 9:**
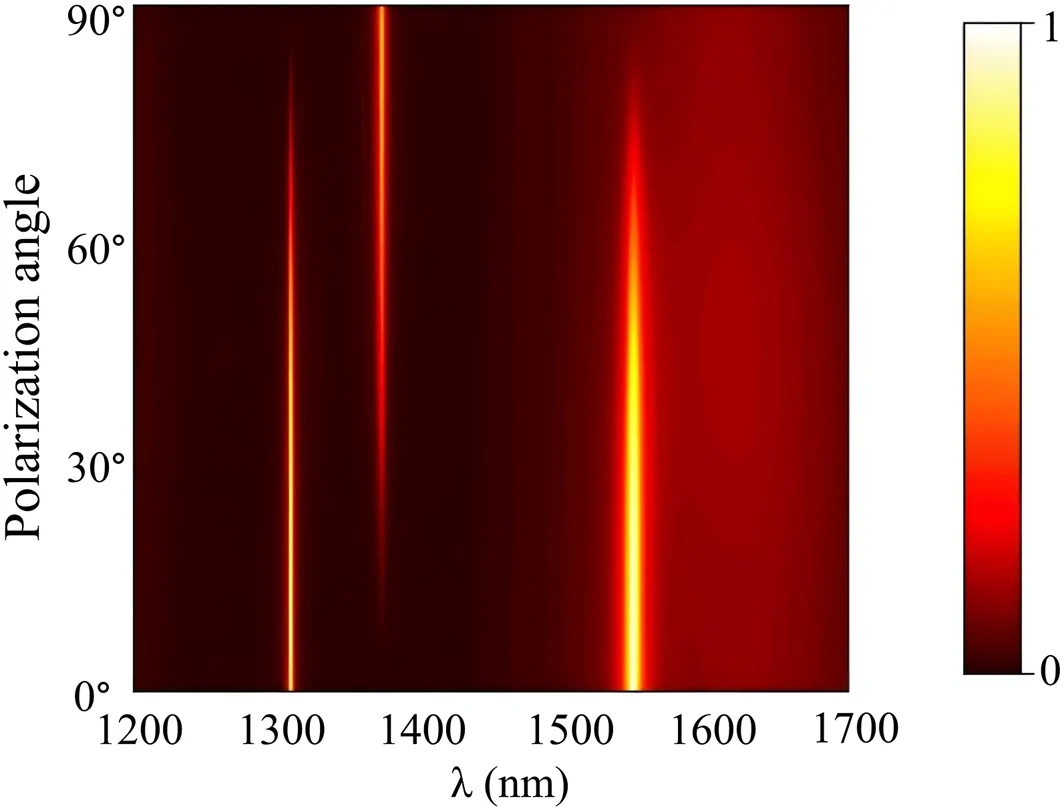
Absorption spectra of the LN metamaterial absorber for different polarization angles.

## 4 All‐Optical Modulation of LN Coherent Perfect Absorber

To further enhance the functionality of LN‐MMA and broaden its applications, we introduce coherent absorption theory to realize an LN‐CPA, whose structural parameters are optimized using the same “end‐to‐end + gradient optimization” framework. To enable coherent absorption, the metal substrate of the MMA is removed, transforming the device from a single‐port absorber into a two‐port interference system. This structure breaks the original impedance matching, making it necessary to reoptimize the key geometric parameters, as schematically illustrated in Figure 10. The period (*P*_*x*_ = *P*_*y*_ = 1000 nm) and grating height *t*_0_ = *h*_0_ = 200 nm) are fixed, whereas the grating width *w’*, LN thickness *t*_1_, and transparent dielectric layer (*n*_3_ = 1.73) thickness *t*_2_ are treated as design variables. A dataset of 5000 samples is generated by Latin hypercube sampling with *t*_1_ ∈ [50, 300] nm, *t*_2_ ∈ [1000, 1300] nm, and *w’* ∈ [400, 700] nm and the spectra were sampled over the wavelength range from 1500 to 1800 nm with a step size of 2 nm. The same data split as that for the LN‐MMA was used. All other parameters remain unchanged. Except for the Au substrate, the LN‐CPA retains the planar multilayer configuration of the LN‐MMA and can therefore follow a similar fabrication process. Experimental implementation requires optical access from both sides and an electrode layout compatible with the two‐port geometry. The relative phase and intensity of the two incident beams must also be stably controlled [25]. The incident beams *I*_1_ and *I*_2_ correspond to output beams *O*_1_ and *O*_2_, respectively. The relationship between the input and output light beams can be expressed as [49]

(10) $$\left[\begin{array}{l} S_{1}\\ S_{2} \end{array}\right]=S\left[\begin{array}{l} E_{1}\\ E_{2} \end{array}\right],S=\left[\begin{array}{cc} S_{11} & S_{12}\\ S_{21} & S_{22} \end{array}\right],$$

where *S*_11_ = *r*_1_exp[*i*(*φ*_11_ + *φ*_0_)], *S*_12_ = *t*_2_exp(*iφ*_12_), *S*_21_ = *t*_1_exp[*i*(*φ*_21_ + *φ*_0_)], and *S*_22_ = *r*_2_exp(*iφ*_22_). Here, *E*_1_, *E*_2_, *S*_1,_ and *S*_2_ are the electric amplitudes of the incident and scattered fields, respectively. *r*_1_, *r*_2_, *t*_1_, and *t*_2_ are the reflection and transmission coefficients of incident beams *I*_1_ and *I*_2_. *φ*_11_, *φ*_12_, *φ*_21,_ and *φ*_22_ are the reflection and transmission phases of *I*_1_ and *I*_2_. *φ*_0_ is the phase difference between *I*_1_ and *I*_2_. The output beams can be written as

(11) $$\begin{array}{l} O_{1}=\frac{Z_{0}}{2}S_{1}^{*}S_{1}=\frac{Z_{0}}{2}\left[{\left(r_{1}E_{1}\right)}^{2}+{\left(t_{2}E_{2}\right)}^{2}+2r_{1}t_{1}E_{1}E_{2}\;\cos\left(\varphi_{11}+\varphi_{0}+\varphi_{12}\right)\right]\\ O_{2}=\frac{Z_{0}}{2}S_{2}^{*}S_{2}=\frac{Z_{0}}{2}\left[{\left(t_{1}E_{1}\right)}^{2}+{\left(r_{2}E_{2}\right)}^{2}+2t_{1}r_{2}E_{1}E_{2}\;\cos\left(\varphi_{21}+\varphi_{0}+\varphi_{22}\right)\right] \end{array},$$

where *Z*_*0*_ denotes the impedance of the structure. The coherent absorption *A*_co_ is expressed as

(12) $$A_{\text{co}}=1-\frac{O_{1}+O_{2}}{I_{1}+I_{2}}=1-\frac{S_{1}^{*}S_{1}+S_{2}^{*}S_{2}}{E_{1}^{*}E_{1}+E_{2}^{*}E_{2}}.$$

**FIGURE 10:**
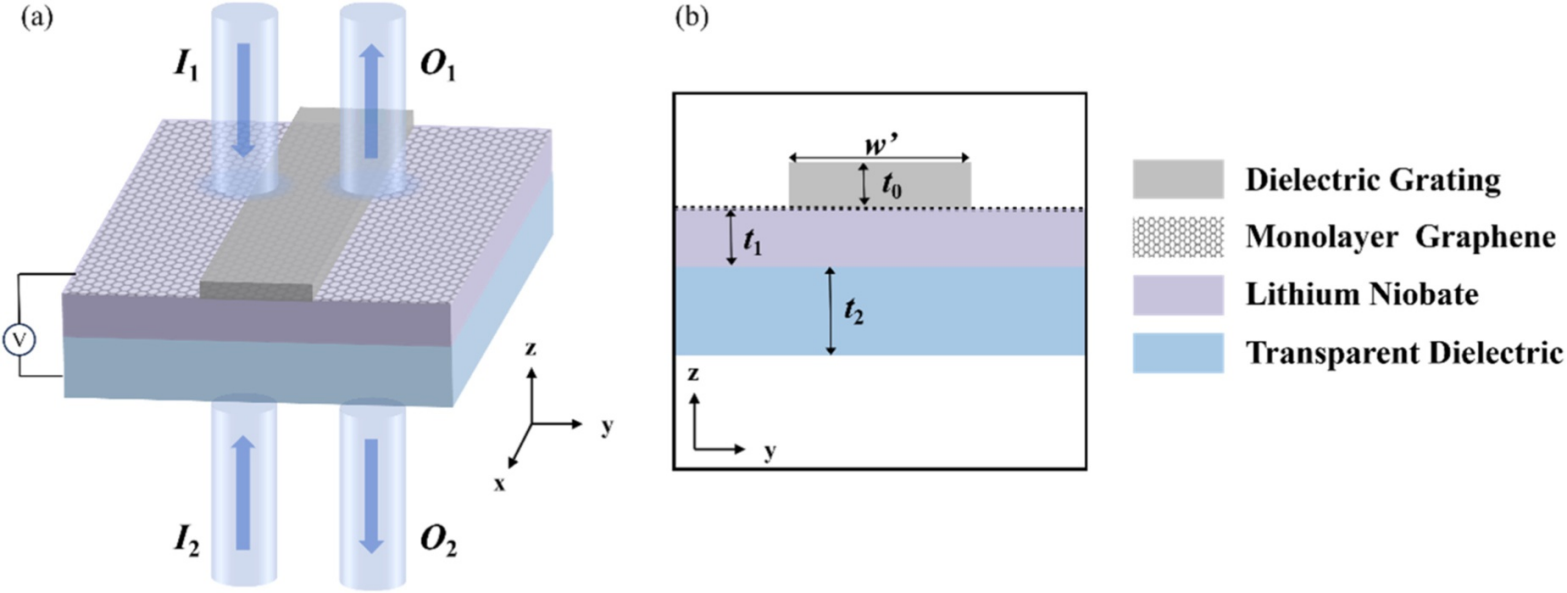
(a) Schematic of the proposed LN‐CPA. (b) Cross‐section view of the proposed LN‐CPA.

From Equation (10), we notice that the CPA occurs when the scattering matrix S reaches zero, which requires

(13) $$r_{1}r_{2}\;\exp\left(\varphi_{11}+\varphi_{22}\right)=t_{1}t_{2}\;\exp\left(\varphi_{21}+\varphi_{12}\right).$$

In addition, considering *r*_1_, *r*_2_, *t*_1_, *t*_2_, *E*_1,_ and *E*_2_ are positive real numbers, *S*_1_ = *S*_2_ = 0 requires that

(14) $$\begin{array}{l} \varphi_{11}+\varphi_{0}=\varphi_{12}+\pi \\ \varphi_{21}+\varphi_{0}=\varphi_{22}+\pi \end{array}.$$

According to Equations (10) and (11), the incident intensity requirement for the CPA is

(15) $$r_{1}\sqrt{I_{1}}=t_{2}\sqrt{I_{2}}\;\text{and}\;t_{1}\sqrt{I_{1}}=r_{2}\sqrt{I_{2}}.$$

Equations ((13), (14), (15)) give the structural requirements (*r*_1_, *r*_2_, *t*_1_, *t*_2_, *φ*_11_, *φ*_12_, *φ*_21_, and *φ*_22_) and incident requirements (*I*_1_, *I*_2_, and *φ*_0_) for asymmetric CPA.

The training performance of the forward surrogate model of LN‐CPA is shown in Figure 11. There is no significant difference between the training loss (1.50 × 10^−3^) and the validation loss (1.26 × 10^−3^), indicating that the forward surrogate model does not overfit. Figure 11b,c shows the comparison of spectral prediction accuracy among different surrogate models, demonstrating the superior performance of our forward surrogate scheme in similar tasks. Based on the well‐trained forward model, inverse design and gradient optimization are performed to realize a dual‐band coherent absorber with near‐perfect absorption at 1550 and 1704 nm. During the simulation process, the amplitude ratio of the two incident beams is fixed as *I*_2_ = 1.2 *I*_1_ to define the coherent excitation condition. The inverse design and gradient optimization results are shown in Figure 12. The training loss (2.79 × 10^−2^) closely matches the validation loss (2.76 × 10^−2^), indicating no overfitting. As seen from the ablation results in Figure 12b, our configuration still outperforms MLP‐NR and KAN‐NR, demonstrating that the proposed “end‐to‐end + gradient optimization” framework is effective across different task scenarios, thereby demonstrating its robustness and versatility for metasurface design. The optimized structural parameters corresponding to each framework are summarized in Table 3.

**FIGURE 11:**
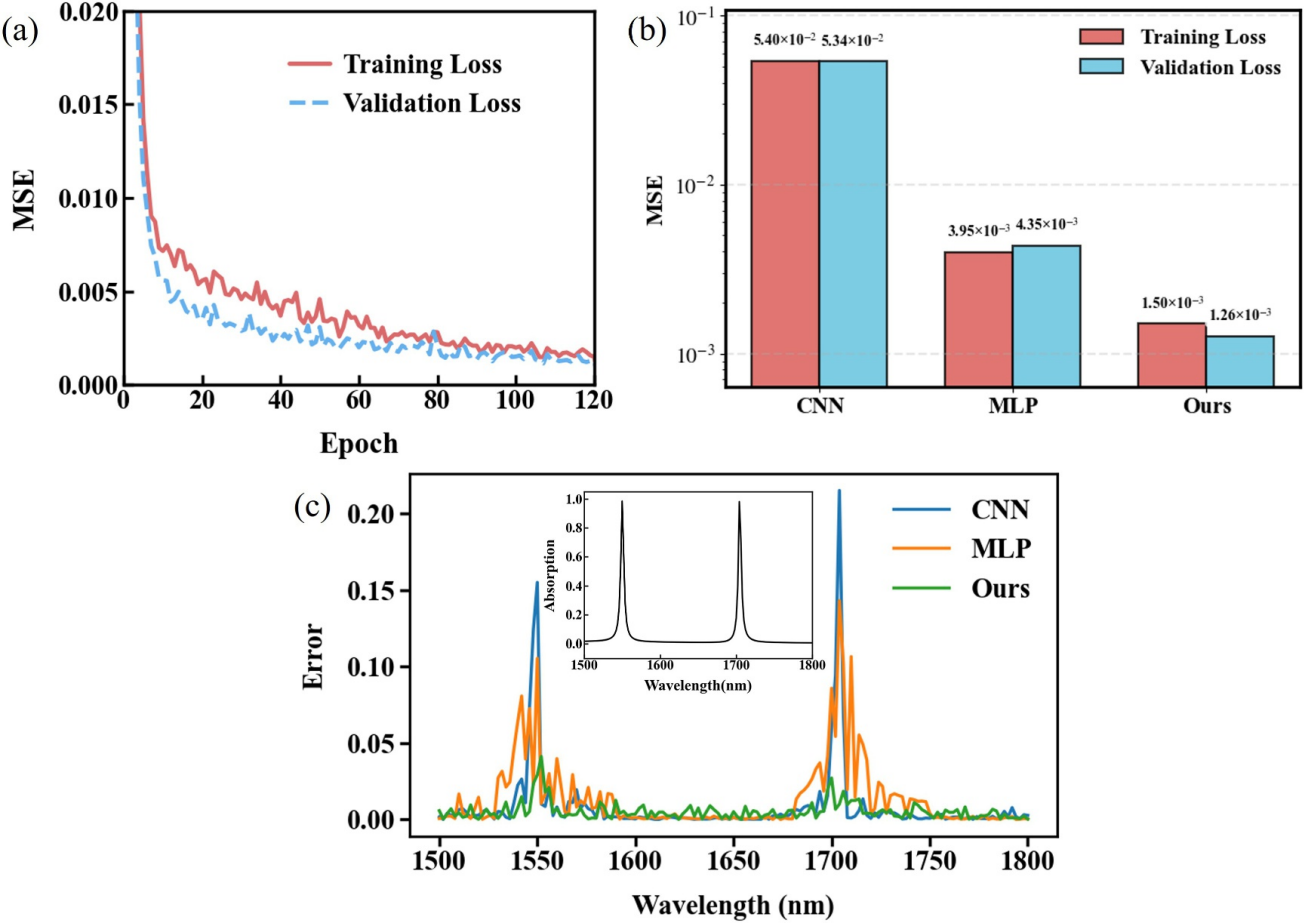
Training performance of the forward surrogate model of LN‐CPA. (a) The training and validation loss curves during the training process. (b) Loss values for different algorithms, including CNN, MLP, and our proposed method. (c) Prediction errors for different methods across the wavelength range. The inset shows the actual absorption spectrum.

**FIGURE 12:**
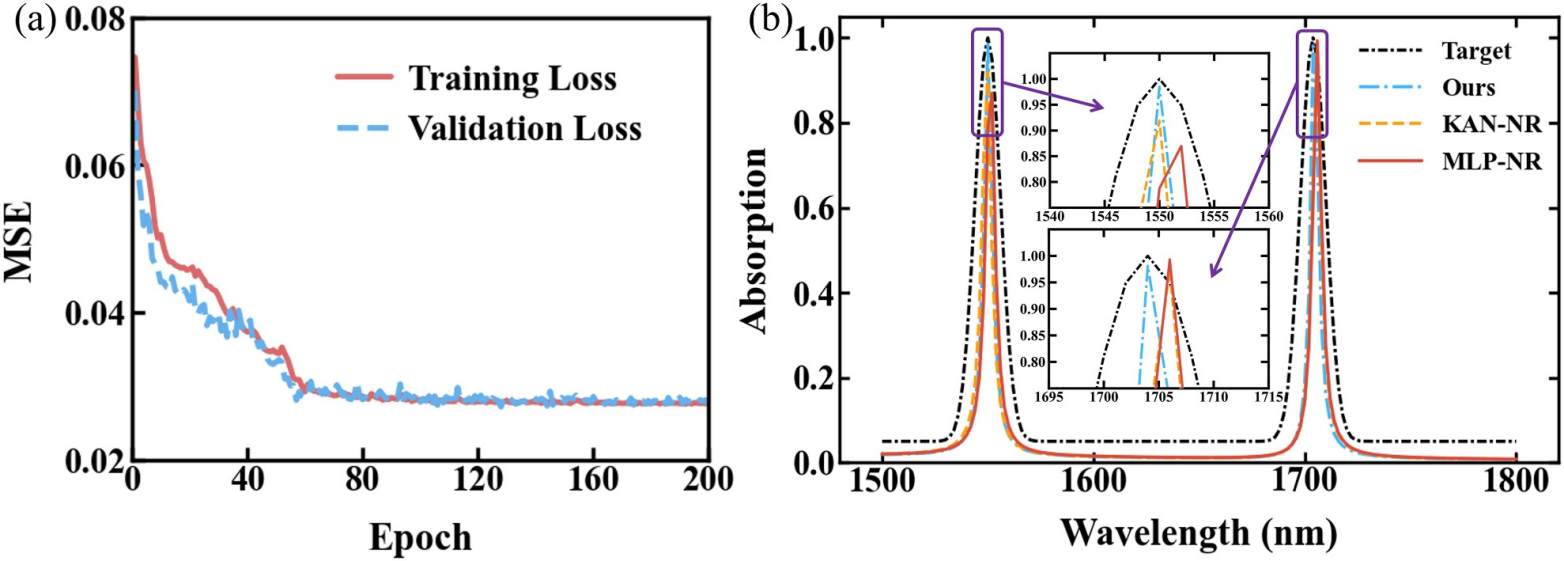
(a) Total loss of the inverse design model. (b) Ablation experiment: Absorption spectra corresponding to different schemes of CPA design.

**TABLE 3:** Results of different LN‐CPA design schemes.

Inverse design solution	*t*_1_ (nm)	*t*_2_ (nm)	w’ (nm)
MLP‐NR	142.35	1120.03	563.33
KAN‐NR	142.37	1111.42	563.45
Ours	139.98	1120.00	640.02

To further evaluate the computational efficiency of the proposed framework, the computational costs are summarized in Table 4. All wall‐clock times were measured in the CPU‐only environment described in Section 2.2. The training times were averaged over 10 independent runs, whereas the target‐specific design times were averaged over 1000 target spectra and included both the initial inverse prediction and gradient refinement. After training, the final structural parameters were obtained within a few seconds without additional FEM simulations.

**TABLE 4:** Computational costs of network training and target‐specific inverse design.

Device	Forward surrogate training (min)	Inverse network training (min)	Total training time (min)	Target‐specific design time (s)
LN‐MMA	16.2	45.6	61.8	2.3
LN‐CPA	17.3	48.4	65.7	2.8

To assess dataset adequacy, we compared the test performance using 2000–5000 samples while keeping the same 500 validation samples and 500 test samples. The forward error was calculated against the FEM spectra, whereas the predicted structures were re‐simulated using FEM for inverse‐design evaluation. As shown in Figure 13, the test errors decrease as the dataset size increases for both LN‐MMA and LN‐CPA. Since each task contains only three bounded structural parameters, 5000 samples provide adequate coverage and meet the accuracy requirements of the present design tasks.

**FIGURE 13:**
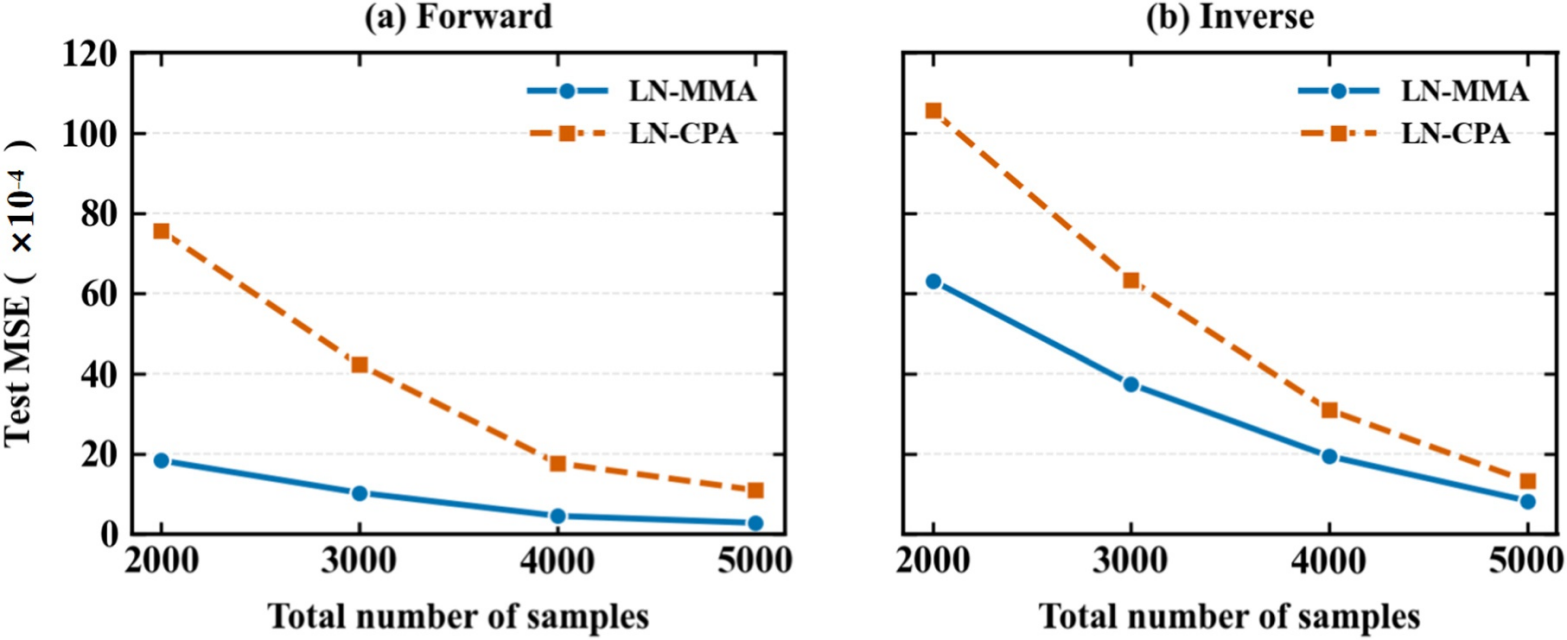
Test MSE of LN‐MMA and LN‐CPA for different dataset sizes: (a) forward prediction and (b) inverse‐design evaluation. The validation and test sets each contained 500 samples.

Figure 14a shows the absorption spectrum of the designed LN‐CPA. When the bias voltage is 0 V, the absorber exhibits two near‐perfect absorption peaks at *p*_3_ = 1550 nm and *p*_4_ = 1704 nm with efficiencies of 98.5% and 98.1%, respectively. Similar to the LN‐MMA, the resonance wavelengths can be dynamically tuned via the electro‐optic effect of lithium niobate. A forward voltage induces a blue shift, whereas a reverse voltage induces a red shift. As shown in Figure 14b, the linear fitting results indicate that the voltage modulation rates of the two absorption peaks are −0.046 nm/V and −0.059 nm/V for *p*_3_ and *p*_4_, respectively. As shown in Figure 14c,d, *I*_2_/*I*_1_ was varied from 0.8 to 1.6 to evaluate the influence of the incident intensity ratio. The two resonance wavelengths remain almost unchanged, and the peak absorptions remain above about 97%. This indicates good tolerance to intensity mismatch. The inverse optimization simultaneously minimizes the deviation from the CPA condition around both target wavelengths. The exact CPA condition is not strictly identical at the two resonances because the complex scattering coefficients are wavelength‐dependent. According to Equation (12), the deviation from the ideal CPA state can be directly quantified by *δ*_CPA_ = 1−*A*_co_. The calculated values are only 0.02361 and 0.02304 at 1550 and 1704 nm, respectively. This confirms that the proposed structure operates in a dual‐band near‐CPA state with low residual scattering. Owing to their narrow full width at half maximum (FWHM), the device provides excellent voltage‐wavelength discrimination at both resonance wavelengths, making it suitable for voltage detection, sensing, and photoelectric signal conversion.

**FIGURE 14:**
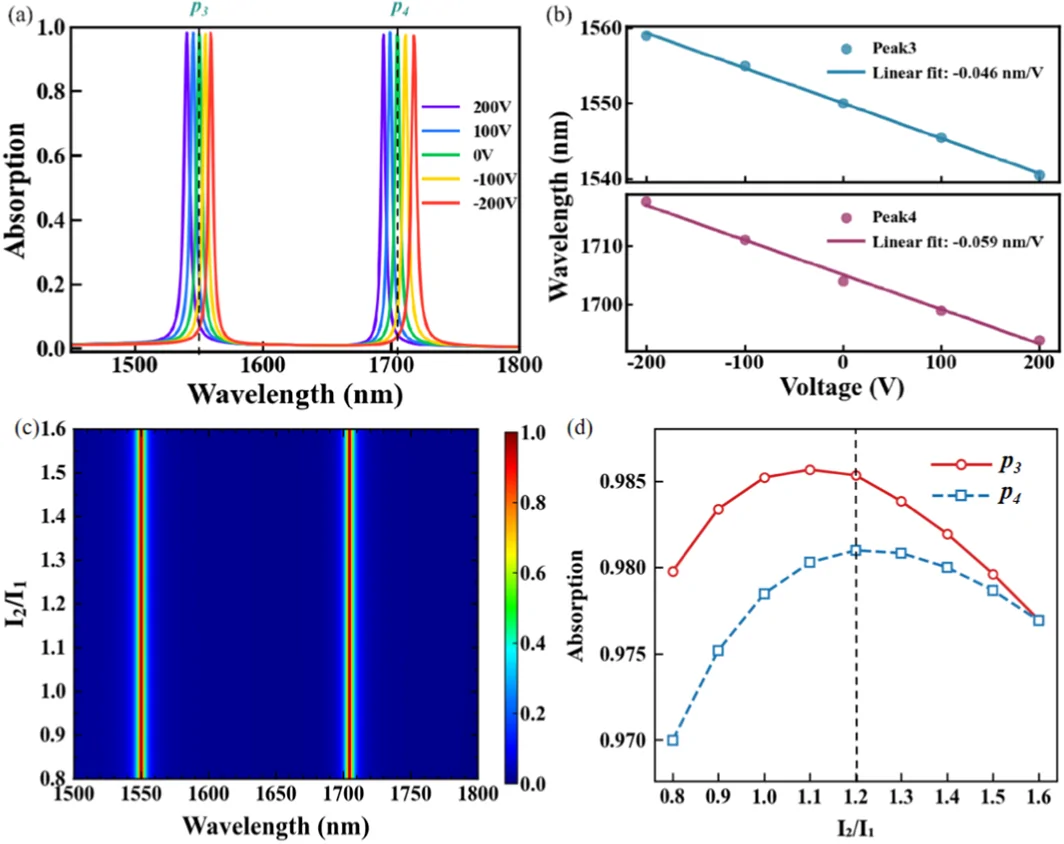
Absorption performance and robustness analysis of the LN‐CPA. (a) Absorption spectra under different applied voltages. (b) Resonance wavelengths of *p*_3_ and *p*_4_ as functions of the applied voltage. (c) Coherent absorption spectrum as a function of the incident intensity ratio *I*_2_/*I*_1_. (d) Peak absorptions at *p*_3_ = 1550 nm and *p*_4_ = 1704 nm as functions of *I*_2_/*I*_1_. The dashed line indicates the design condition *I*_2_/*I*_1_ = 1.2.

To elucidate the physical origin of the dual‐band absorption, the electric field distributions at *p*_3_ and *p*_4_ are shown in Figure 15, indicating both resonances originate from TE‐polarized quasi‐guided mode resonances. At *p*_3_, the field is periodically concentrated along the *y*‐direction in both the LN and dielectric layers, corresponding to a higher‐order TE quasi‐guided mode [50, 51, 52]. In contrast, at *p*_4_, the field is mainly localized at the LN/dielectric interface, implying a composite waveguide mode formed by both layers. Using Equation (8), the theoretical resonance wavelength *λ*_4_ = 1707 nm agrees well with the simulated 1704 nm, further validating the physical model.

**FIGURE 15:**
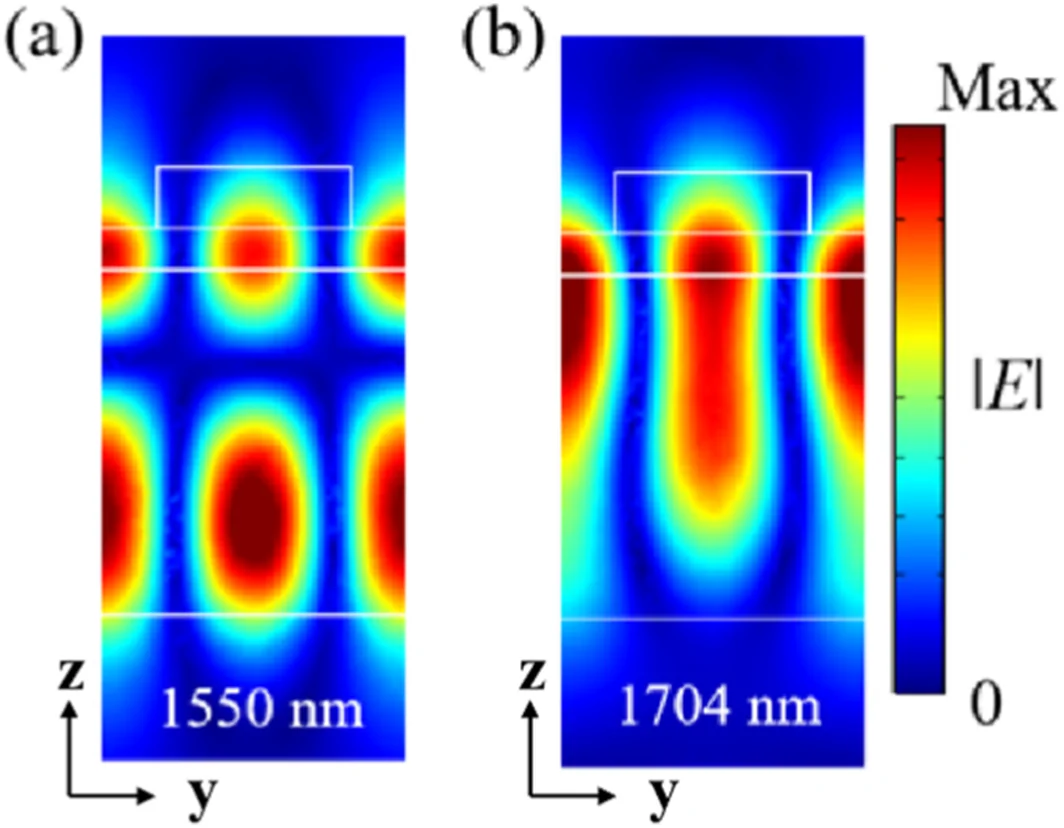
(a) Electric field distribution on the cross‐section view of the LN‐CPA at 1550 nm. (b) Electric field distribution on the cross‐section view of the LN‐CPA at 1704 nm.

To study all‐optical modulation, we analyzed the dependence on the phase difference between two incident coherent beams (Figure 16). The absorptions at *p*_3_ and *p*_4_ exhibit periodic modulation between high absorption and high transmission as the phase difference varies. When the phase difference is zero, both resonances exceed 98% absorption, whereas at a phase difference of π, the absorption at both wavelengths drops below 1%. Thus, the LN‐CPA enables phase‐controlled tuning of absorption from 1% to 98%, functioning as a near‐infrared all‐optical switch. The modulation depths, defined as MD = (*A*_max_–*A*_min_)/*A*_max_ [53], reach 99.1% at 1550 nm and 98.8% at 1704 nm, indicating excellent all‐optical switching performance.

**FIGURE 16:**
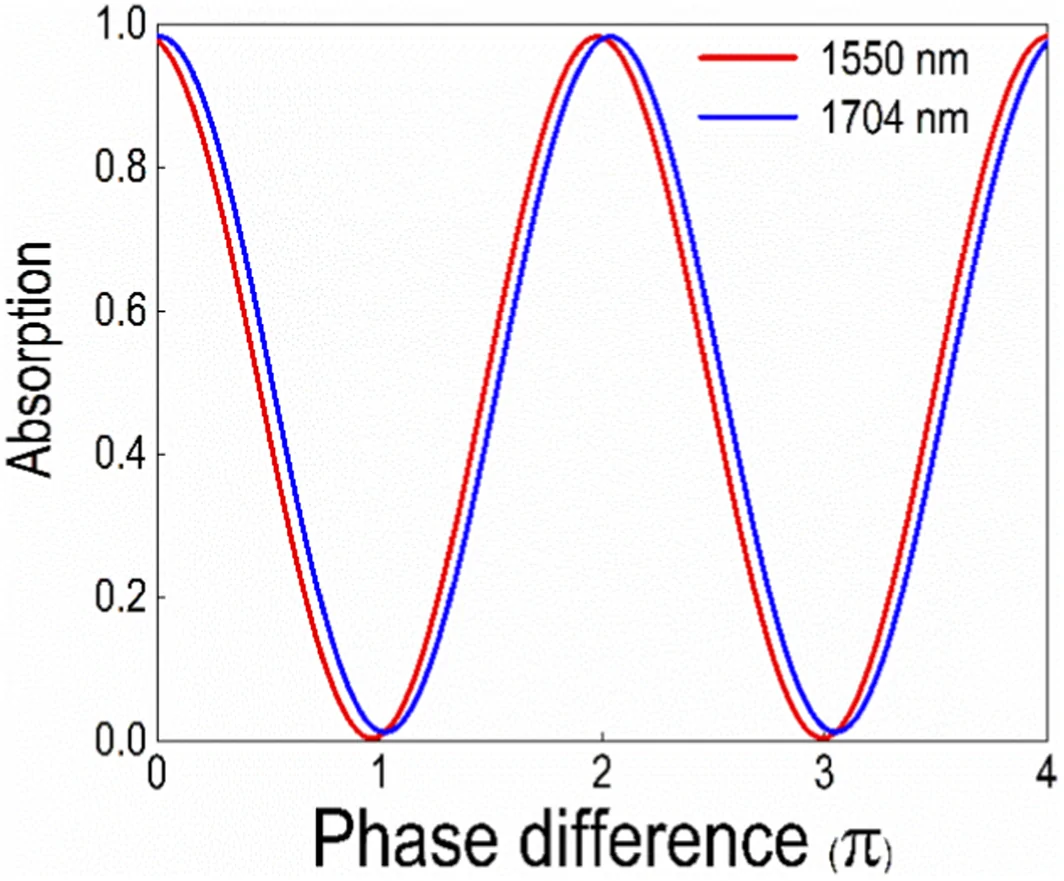
Absorption of LN‐CPA with the phase difference between two incident coherent beams at 1550 and 1704 nm.

The effect of the polarization angle on coherent absorption is shown in Figure 17. For polarization angles between 0° and 30°, the absorption at the two resonance peaks remains nearly unchanged. When the angle exceeds 30°, their absorption gradually decreases, accompanied by the emergence of two additional resonances at 1464 and 1658 nm. At 90° (*y*‐polarization incidence), the original resonances vanish, whereas the new peaks reach absorption efficiencies up to 80%. Therefore, the LN‐CPA maintains robust performance over small polarization angles and still exhibits strong absorption under *y*‐polarized incidence. This behavior indicates a potential polarization‐selective dual‐mode operation. Polarization rotation breaks the original resonant condition. The quasi‐guided modes are supported by the grating‐LN‐dielectric structure. So, the original resonances are suppressed, whereas another set of polarization‐dependent modes 1464 and 1658 nm appear. The corresponding electric‐field distributions in Figure 18 confirm that they are from different quasi‐guided modes.

**FIGURE 17:**
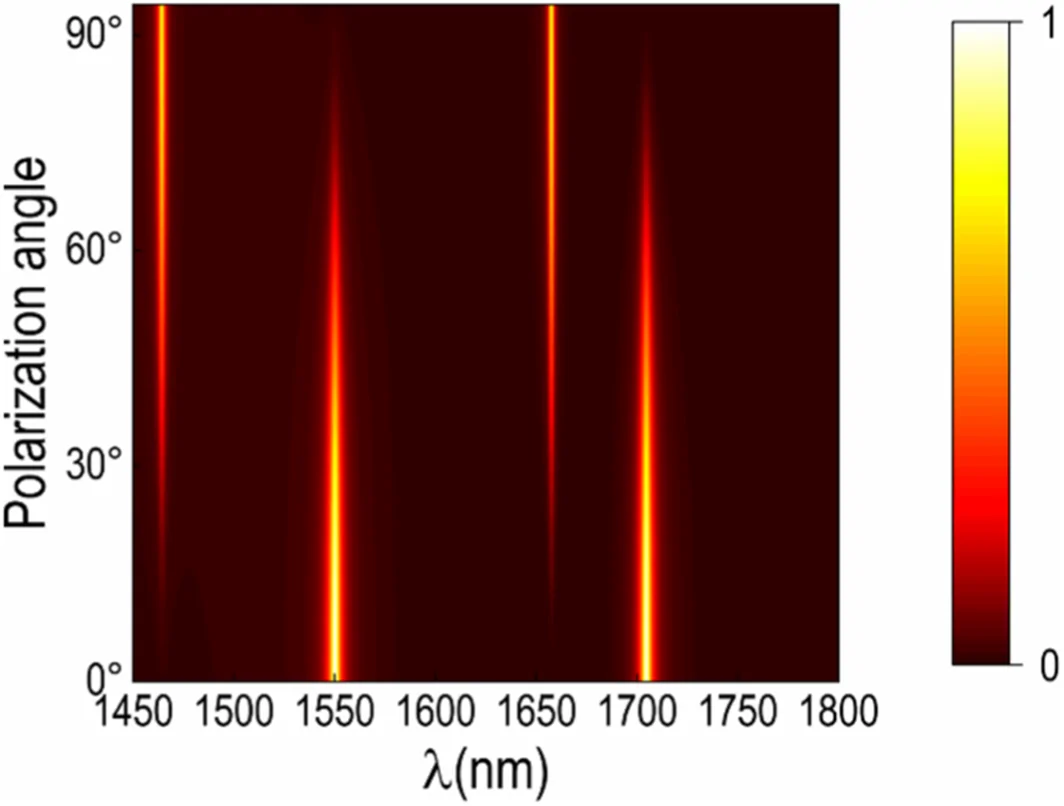
Absorption spectra of LN‐CPA for different polarization angles.

**FIGURE 18:**
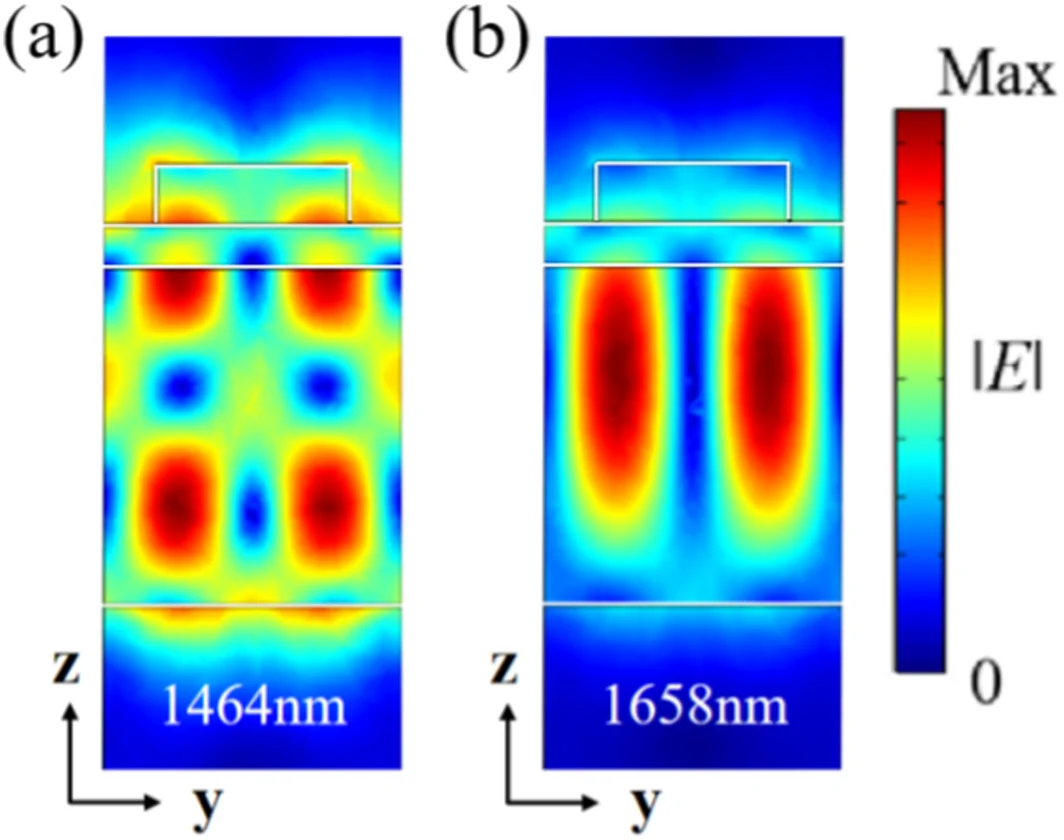
(a) Electric field distribution on the cross‐section view of the LN‐CPA at 1464 nm under *y*‐polarized incidence. (b) Electric field distribution on the cross‐section view of the LN‐CPA at 1658 nm under *y*‐polarized incidence.

To assess the temperature stability of the proposed devices for potential space applications, the absorption spectra were recalculated at 100 K, 298 K, and 750 K, as shown in Figure 19. The temperature‐dependent refractive indices of LN were obtained from Hong et al. [54], with the 100 K values obtained by extrapolation. Other material properties and structural parameters were kept unchanged. The maximum resonance shifts are 0.2 and 1.8 nm for the LN‐MMA and 0.7 and 0.8 nm for the LN‐CPA. These results demonstrate that thermo‐optic variations in LN mainly induce minor resonance wavelength shifts while maintaining the absorption efficiency and spectral characteristics of the proposed devices over a wide temperature range, indicating excellent thermo‐optic stability and highlighting their potential for space optical communication applications.

**FIGURE 19:**
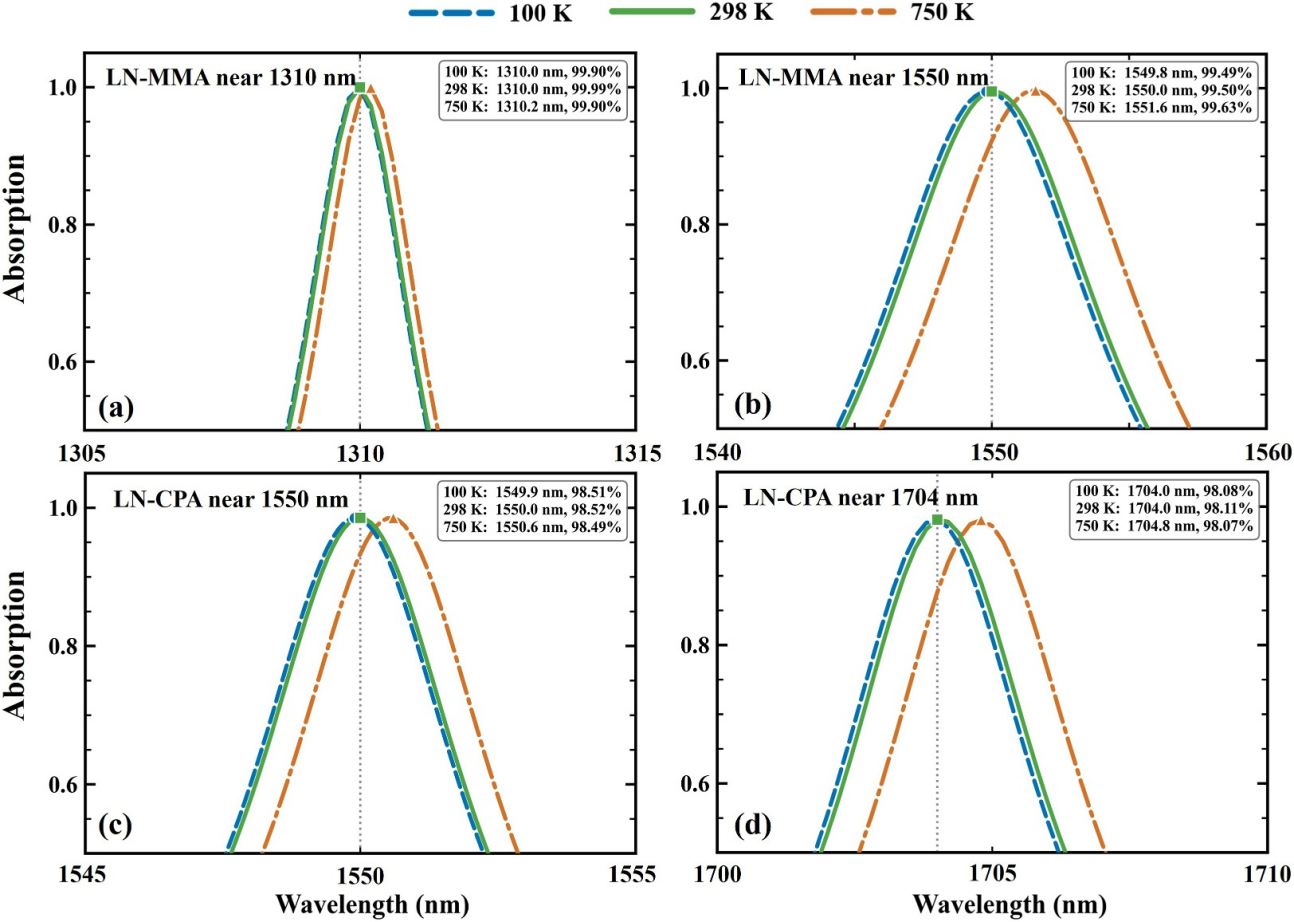
Temperature‐dependent absorption spectra of the proposed devices at 100 K, 298 K, and 750 K: (a, b) LN–MMA and (c, d) LN–CPA. The dotted lines indicate the resonance positions at 298 K.

Finally, the absorption wavelength, absorption efficiency, and tuning method of the designed LN metamaterial absorber are summarized and compared with recently reported studies, as shown in Table 5. The comparison shows that the proposed LN metamaterial absorber in the communication band exhibits competitive overall performance and uniquely combines dual‐band operation with both electrical and all‐optical tunability.

**TABLE 5:** Comparison of communication band absorber between our work and previous reports.

Reference	Absorption wavelength (nm)	Absorption efficiency	Tuning method
[11]	1550	99%	—
[55]	1310/1550	99.9%/99.4%	—
[56]	1310 & 1550	99.8% & 99.5%	—
This work	1310 & 1550	99.9% & 99.5%	All‐optical & electric

## 5 Conclusion

In summary, we have proposed and analyzed a dynamically tunable near‐infrared dual‐band LN metamaterial absorber platform at optical communication wavelengths. The first device is a dual‐band LN‐MMA, which shows near‐perfect absorption at 1310 and 1550 nm, and supports continuous electrical tuning of the resonance wavelengths through the EO effect of LN. The second device is an LN‐CPA designed to support near‐perfect absorption at 1550 and 1704 nm. It realizes high contrast all‐optical modulation by adjusting the phase difference between two coherent input beams. The structural parameters of the devices are obtained using an “end‐to‐end + gradient optimization” deep‐learning‐based inverse‐design framework, which serves as an efficient numerical tool for matching the target spectra in a high‐dimensional parameter space. As the present results are simulation‐based, future experimental work should address fabrication tolerances, electrode integration, coherent‐beam stability, temperature‐induced resonance shifts, and environmental stability under space‐relevant conditions. Overall, the LN‐MMA and LN‐CPA offer compact, dual‐band, and dynamically tunable platforms with potential applications in inter‐satellite laser communication, photoelectric signal processing, voltage sensing, and all‐optical switching. In addition, the proposed deep‐learning‐assisted design framework can be extended to other photonic devices and wavelength ranges.

## Author Contributions

**Xiajun Liu:** writing – original draft, software, methodology, data curation, conceptualization. **Zexuan Zhang:** writing – original draft, data curation, methodology. **Chenxi Su:** writing – original draft, methodology, data curation. **Mei Wang:** validation. **Feng Xia:** validation. **Peng Sun:** writing – review and editing. **Li Dong:** writing – review and editing, validation. **Maojin Yun:** writing – review and editing, validation, supervision.

## Funding

This work was supported in part by the Natural Science Foundation of Shandong Province, China (ZR2024QF074, ZR2020MF108), and in part by the National Natural Science Foundation of China (U25A20509).

## Conflicts of Interest

The authors declare no conflicts of interest.

## Data Availability

Data available on request from the authors.
